# Some Characteristics and Arguments in Favor of a Science of Machine Behavior Analysis

**DOI:** 10.1007/s40614-022-00332-3

**Published:** 2022-03-31

**Authors:** Marc J. Lanovaz

**Affiliations:** 1 École de psychoéducation, Université de Montréal, C.P. 6128, succursale Centre-Ville, Montreal, QC H3C 3J7 Canada; 2 Centre de recherche de l’Institut universitaire en santé mentale de Montréal, Montreal, Canada

**Keywords:** Artificial intelligence, Behavior analysis, Machine learning

## Abstract

Researchers and practitioners recognize four domains of behavior analysis: radical behaviorism, the experimental analysis of behavior, applied behavior analysis, and the practice of behavior analysis. Given the omnipresence of technology in every sphere of our lives, the purpose of this conceptual article is to describe and argue in favor of a fifth domain: machine behavior analysis. Machine behavior analysis is a science that examines how machines interact with and produce relevant changes in their external environment by relying on replicability, behavioral terminology, and the philosophical assumptions of behavior analysis (e.g., selectionism, determinism, parsimony) to study artificial behavior. Arguments in favor of a science of machine behavior include the omnipresence and impact of machines on human behavior, the inability of engineering alone to explain and control machine behavior, and the need to organize a verbal community of scientists around this common issue. Regardless of whether behavior analysts agree or disagree with this proposal, I argue that the field needs a debate on the topic. As such, the current article aims to encourage and contribute to this debate.

Since Skinner initially proposed a science of behavior in the 1930s, the world in which humans live has evolved tremendously. One of the most notable changes is the pervasive presence of technology in our homes and our workplaces. To put things in perspective, researchers only built the first automatic digital computers in the 1940s (Watson & Calhoun, 1960). These computers needed large rooms and could only conduct basic calculations to solve scientific problems. Skinner experimented with machines (albeit nondigital) such as the air crib, the teaching machine, and the operant conditioning chamber (i.e., often referred to as a Skinner box) during his lifetime (Skinner, 1958, 1961). He even discoursed on whether humans and machines were really that different (Skinner, 1969). However, this early technology does not compare to the diversity of functions that contemporary computers can carry out. Nowadays, smartphones can not only fit in our pockets, but they are also significantly more powerful than early computers. As an example of the omnipresence of technology in our lives, 84% of Americans with a smartphone report consulting it within 15 min of getting up in the morning (Levitas, 2013).

In the past decades, the development and the application of new algorithms (i.e., sets of computer instructions that solve a problem) as well as progress in computing power have allowed machines to reach a point wherein electrical and computer engineers^1^ are oftentimes unable to predict how machines will “respond” given an input (Rudin, 2019; Sendak et al., 202l; Watson et al., 2019). In an example popularized by the media, Microsoft developed a Twitter chatbot, named Tay, which was supposed to learn to hold conversations online. After only 24 hr, the development team had to step in because Tay had learned “to tweet like a Nazi sympathizer, racist and supporter of genocide, amongst other things” (Wakefield, 2016). Based on their knowledge of machine learning algorithms and coding, the engineers could not predict Tay’s behavior once left to fend on its own “in the wild.” In this example, studying the behavior of the machine (i.e., a chatbot) when presented with different inputs may have prevented this unfortunate incident. This article aims to argue that the field needs a science of machine behavior analysis to address this issue and many others that stem from rapid technological developments.

At present, behavior analysts typically recognize four domains of behavior analysis: radical behaviorism, the experimental analysis of behavior, applied behavior analysis, and the practice of behavior analysis (Cooper et al., 2020; Moore, 2008). The focus of these domains is humans and nonhuman living organisms. Behavior analysis does not have a domain that focuses on the responding of machines to their external environment. The main thesis of this conceptual article is that behavior analysts need to formalize a fifth domain to address this issue: machine behavior analysis.

## Some Basic Characteristics

Before making an appeal for the formalization of a science of machine behavior analysis, the first step is to establish its potential boundaries. In the current section, I describe the five basic characteristics of a science of machine behavior analysis. These characteristics should not be perceived as complete or exclusive, but rather as a starting point to better define the science and spur discussions.

### Centered on Machine Behavior

As indicated in the introduction, the main distinction of machine behavior analysis is its emphasis on machines. Rather than directing its efforts towards the behavior of humans or other living organisms, the science focuses on machine behavior. The logical question that follows is: “What should be considered a machine?” Unfortunately, this question does not have a straightforward answer. For example, the *Merriam-Webster.com* dictionary contains no fewer than 12 current definitions of machine, one of which refers to living organisms. It is clear that the current article should not focus on naturally occurring living organisms, which is already the purview of the experimental and applied sciences of behavior analysis. Instead, machine behavior analysis involves fabricated apparatuses that produce an observable change in the environment following the presentation (or absence) of certain external events or stimuli while relying on retained system changes.

Whereas experimental and applied behavior analysis focus on the (natural) behavior of living organisms as dependent variables, machine behavior analysis should focus on machine behavior. I use the expression “machine behavior” because behavior on its own is already well-defined. That is, the term “behavior” implicitly applies to living organisms whereas “machine behavior” can be reserved for machines. A simple definition of machine behavior would involve any observable change in the environment produced by a machine. When conducting machine behavior analysis, scientists should focus on the behavior of the machine rather than on the behavior of the living organism. Human behavior still plays a role in a science of machine behavior analysis as it may serve as the independent variable. For example, how does a machine respond to the changes in the environment produced by the human experimenter? In this case, behavioral researchers manipulate human behavior and examine its effects on machine behavior. This approach is not unlike the other domains of behavior analysis: the main change is that the locus of analysis is now the machine. Some situations may also involve both the behavior of the human and the behavior of the machine being studied simultaneously. This type of translational study would involve both applied and machine behavior analysis to identify relations between human and machine behavior.

**Fig. 1:**
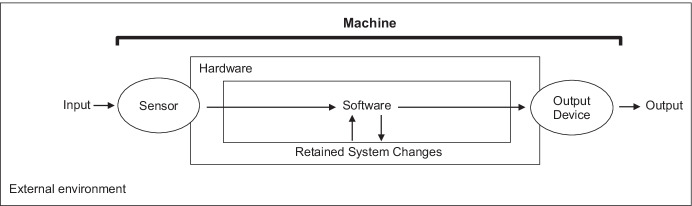
Diagram of a Simplified Machine

Figure 1 presents a diagram of a typical machine that may provide meaningful analyses for behavior analysts. Most machines involve two main components: hardware and software. Hardware is the physical apparatus that runs algorithms such as computers, smartphones, cars, smart speakers, and servers. Software (sometimes referred to as firmware in certain devices) includes the instructions, or algorithms, that tell the hardware what to do. The two components, hardware and software, interact and dictate what a machine can and cannot do. For example, the hardware controls what type of input a machine may receive from its external environment whereas the software uses this data to transform the environment within the limits set by the hardware. Hence, two machines composed of different hardware, but with the same software, may still produce the same behavior. A calculator, a smartphone, and a desktop computer can all multiply numbers efficiently and produce an output of the response on a screen (i.e., they are functionally equivalent). In the same vein, the same hardware may run two different software (e.g., a computer may run a calculator and a music player). In the latter case, scientists would treat each as a different machine process as their functions differ.

In a typical antecedent-behavior-consequence analysis of machine behavior, the antecedent component involves the external environment providing an input to the machine, which is captured by the hardware (see Figure 1). This antecedent may be any environmental change that a machine perceives with its sensors (part of the hardware). Examples include tactile (e.g., keyboard), visual (e.g., camera), auditory (e.g., a microphone), spatial orientation (e.g., gyroscope) and thermal (e.g., heat sensor) inputs. Next, the software uses the data collected by the hardware to determine the output. During this step, algorithms will use part or all of the input data to trigger a signal as output. This process is analogous to the function of the brain in a living organism. Based on this signal, the hardware will typically produce a human-readable output, which can be referred to as a machine behavior in our three-term contingency. Outputs may include movements (e.g., a robot moving its arms), light (e.g., an image on a screen), sounds (e.g., a voice from a smart speaker), and heat (e.g., a heater). Finally, more complex learning machines will record changes produced by their behavior on their environment (i.e., the consequence component). If the machine has the hardware to detect them, the environmental changes produced by the machine (i.e., output) may function as setting events and consequences, which will affect future responding. Machine behavior may thus be amenable to similar analyses as the behavior of learning organisms.

The current article will mainly discuss and provide examples of fabricated nonbiological machines. Researchers have developed robots based on synthetic biological systems (e.g., Blackiston et al., 2021; Kriegman et al., 2020). This reality blurs the distinction between fabricated machines as defined in the current article and living organisms, which may no longer be considered as mutually exclusive (Deplazes & Huppenbauer, 2009). The characteristics and arguments in favor of a science of machine behavior analysis may also apply to organisms developed using synthetic biology (see Abramson & Levin, 2021).

### Relevant

From a behavior analytic perspective, not all machine behavior is relevant to study. The problem is that a machine may produce observable changes (machine behavior) that have limited relevance for behavioral researchers. For example, machine learning may project sets of numbers used by the algorithms on a computer screen. Likewise, interacting with a machine may produce observable changes in electric current. Both these machine behaviors can be observed by the behavioral researcher, but I would argue that neither has a high relevance to them. In the same manner as the brain is the topic of physiological research, the internal state of the machine should remain the realm of engineers. To be relevant to a behavioral scientist, machine behavior should produce an environmental change designed to function as a specific antecedent or a consequence for its user; this user could be a human, a living organism or even another machine. For example, electric current and equations (even though observable) are not designed to function as antecedents and consequences for a layperson using Facebook. As such, the machine behavior could be labeled as irrelevant. Instead, relevant machine behavior may involve Facebook showing you a specific ad on your smartphone, recommending a new friend on a computer, or ordering the posts of your friends on a tablet.

A second dimension of relevance involves the predictability of the machine behavior. When I press on the letter “k” on the keyboard of a computer, the letter “k” always appears on my screen. Although the appearance of “k” on my screen is a machine behavior produced by some external event, its predictability makes its relevance very limited for behavioral researchers. If an algorithm produces responses with a known distribution (e.g., a random number generator returns values from a normal distribution), scientists already know what to expect if the process is repeated for a large number of trials. In other words, a machine behavior is only relevant to study if scientists do not know a priori what specific patterns of responding should be observed. To return to the Microsoft experiment, an example of relevant machine behavior is how Tay, the chatbot, changes the content of its tweets based on different human behavior. The programmers themselves were unaware how the external environment (i.e., the tweets of others) would artificially shape its tweeting behavior. A behavior-analytic approach would have been well-suited to study this type of issue.

### Replicable

To ensure progress, any science should be replicable and machine behavior analysis makes no exception. Consistent with the dimensions of applied behavior analysis (Baer et al., 1968), a replicable science should make use of technological descriptions. Researchers must describe their procedures in sufficient detail so that someone with training in the science could replicate their methodology. Another dimension of replicability involves using research methodologies that can produce reproducible results. The science should strive to show prediction and control over machine behavior. Most machines that engage in relevant behavior probably have their own idiosyncratic responding because they may have different initial conditions (e.g., random number matrices to initiate model), different histories of contact with their environment, or both. Thus, single-case methodology may play a central role in the development of the science (Kazdin, 2021). Single-case designs may not only facilitate within- and between-subject replications, but they may also examine idiosyncratic responding across machine subjects.

For example, let’s suppose that we want to study how a chatbot, that takes auditory and visual stimulation as input, adapts its machine behavior to its social environment. In a typical situation, engineers would use simulations by providing videos as input during training and testing to observe how the machine reacts and adapts. However, these simulations may not perfectly mimic the type of interactions that chatbots have in nonsimulated environments, making it difficult to predict how they will respond in different conditions (think of Tay here). To support their development, behavioral researchers may work with engineers to test the chatbot in the real-world and recommend modifications. To examine how this chatbot learns new behavior, a behavioral researcher may use a basic reversal design. In Phase A, the experimenter may say a made-up word on a time-based schedule while maintaining a neutral facial expression. In Phase B, the experimenter may say the same made-up word and smile whenever the robot engages in a machine behavior (e.g., moving, saying “hi”) within 2 s of the stimulus presentation. This process would be akin to establishing stimulus control in a living organism by using positive reinforcement (if smiling were programmed as a reinforcer). Then, the teaching parameters may be manipulated (e.g., delay prior to reinforcement, schedule of reinforcement) to see how the robots adapts within an alternating treatment design to conduct a parametric analysis. Alternatively, these processes may be repeated in different environments or with chatbots with various learning histories using a multiple baseline design to examine the generalizability of the findings. These analyses would provide unique and valuable information that would be difficult to obtain using simulations in isolation, which underlines the importance of interdisciplinarity in this type of research. Studying machine behavior in this manner, rather than conducting observational studies following release (as done with Tay the chatbot), may prevent unintended consequences for the end users.

One concern regarding replicability is that each machine is programmed in a unique manner. Thus, replicating the same results across different machines may be a challenge. Behavior analysts are already aware of this issue because each species, as well as each individual within a species, is unique. For individuals within species, variations in responding may be explained by variations in the initial conditions. These initial conditions include organism-specific genes and prior contact of the individual organism with the environment over which the experimenter has no control. These variations are even larger across species because different species have distinct genes. Therefore, algorithms could be viewed as species. Each use of the same algorithms only differs in its starting condition (i.e., data provided by the experimenter and data extracted from the environment). Some general rules may emerge from studying the same algorithm, which may lead to replicable experimental results. In contrast, studying different algorithms could be viewed as studying the behavior of different species. Given their emphasis on the study of the behavior of *individuals*, behavioral researchers should apply their expertise to this novel class of subjects (i.e., machines).

### Consistent with Behavior Analytic Terminology

To allow communication between scientists working in different domains, a science of machine behavior analysis should remain terminologically consistent. For example, assume that a robot was designed to respond to visual and auditory stimuli. When the robot perceives a human in its environment, it says “hello,” and the human interacts with it. When this same robot perceives a rat in the environment (in the absence of a human), the robot says “hello,” but this is never followed by a subsequent interaction. This robot eventually learns to say “hello” *only* when it perceives a human in its environment. If behavioral researchers applied consistent terminology to the previous example, they could describe the presence of a human in the environment as a discriminative stimulus for the robot engaging in the behavior of saying “hello,” and the human interacting with the robot as a positive reinforcer for this same machine behavior.

The prior example shows one of the challenges of using consistent terminology. Even though the same terms (e.g., “discriminative stimulus,” “reinforcement”) are used, the internal learning mechanisms may differ significantly between living organisms and machines. By keeping the terminology consistent, it may seem that this characteristic is introducing a cognitive bias to the analyses by anthropomorphizing machines. Given that behavioral terms simply describe the impact of environmental variables on behavior (rather than explain internal mechanisms), the terminology remains an accurate description of what is being observed. This is why behavioral researchers may use the same terminology to describe learning across humans and other living organisms. Hence, the application of behavior analytic terminology circumvents this potential cognitive bias.

Another argument in favor of keeping terminology consistent is that others have already appropriated behavioral terminology to describe similar procedures. For example, engineers use the expression “reinforcement learning” when describing a process wherein a machine is more likely to engage in responses that will lead to a preferred outcome (i.e., this preferred outcome is programmed). Abramson and Levin (2021) also proposed using behaviorist terms to study synthetic living organisms. Whenever the use of terms could be misleading, preceding the behavior analytic term with “machine” may provide clarification (e.g., machine behavior, machine reinforcement, machine shaping). Adopting this approach in ambiguous contexts may prevent misleading verbal communities about the processes that scientists are referring to.

### Grounded in the Philosophical Assumptions of Behavior Analysis

As argued since the beginning of this article, I suggest that machine behavior analysis be considered as a fifth domain of behavior analysis. As such, sharing philosophical assumptions with the other domains of behavior analysis appears essential to the development of a coherent and systematic science. Providing a detailed description of the philosophical underpinnings of behavior analysis is beyond the scope of this article, but I refer the reader to Moore (2008) for an introduction to the topic. Nonetheless, the article will draw parallels with some of the philosophical assumptions that behavior analysts contact through their initial training: selectionism, determinism, empiricism, parsimony, and pragmatism (Behavior Analyst Certification Board [BACB], 2017).

Machine behavior analysis adheres to selectionism by relying on the external environment as an explanatory variable. The environment selects the behavior of the machine in the same manner as consequences select the behavior of living organisms. Computer code obviously mediates machine behavior, but this area of research is left to engineers. The main interest of behavioral researchers is how the external environment affects machine behavior. As with the behavior of living organisms, the environment determines the behavior of machines (i.e., determinism). With sufficient experimentation and access to all initial conditions of a machine, scientists should be able to predict machine behavior. As with the study of living organisms and other complex sciences (e.g., weather prediction), the problem is that it may be difficult to consider all starting conditions and their subsequent effects on machine behavior, which limits its predictability in practice. Empiricism involves the assumption that the only way to study a phenomenon is through contact with the environment (Marr, 2008). Therefore, machine behavior analysis relies on human senses, and not on thought experiments, to observe and manipulate the environment to produce meaningful changes. This adoption does not mean that thought experiments cannot be used as a starting point for novel ideas, but that the only way to develop a coherent and replicable science is through contact with the environment.

A science of machine behavior analysis adheres to parsimony. Parsimony justifies the selection of one theory, or concept, over another as follows: “Where we have no reason to do otherwise and where two theories account for the same facts, we should prefer the one which is briefer, which makes assumptions with which we can easily dispense, which refers to observables, and which has the greatest possible generality” (Epstein, 1984, p. 119). Machine behavior analysis should strive to develop a parsimonious science to explain machine behavior by minimizing assumptions. Parsimony may prevent the development of unnecessary concepts to explain machine behavior. For example, assume that a machine is learning to greet someone online to help them with a problem. An observer notices that over time the machine selects greetings in a manner that optimizes the time that the person spends online. A parsimonious explanation may be that the machine selects its greeting based on its prior experience in similar situations, which have been associated with interactions of longer durations. A nonparsimonious explanation would be that the machine has developed self-awareness, which leads it to select an appropriate greeting. The latter concept is less parsimonious as it requires more assumptions (e.g., the existence of self-awareness) than the initial explanation that relies exclusively on the observable environment.

Philosophers have developed many different versions of pragmatism since the late 19^th^ century, which can make it difficult to define (Bacon, 2012; Lattal & Laipple, 2003). Despite being a key concept taught to future practitioners of the science (BACB, 2017), behavior analysts do not necessarily agree on what being pragmatic means (Barnes-Holmes, 2000; Leigland, 2003; Moore, 2016; Schoneberger, 2016). This debate centers around the place of reality and truth within pragmatism as applied to behavior analysis. Nevertheless, a basic premise of pragmatism is that “the true value of a statement is a function of how well the statement promotes effective action” (Moore, 2008, p. 400). A pragmatic science values the extent to which it can control nature or the environment. In applied behavior analysis, this pragmatism transpires through its emphasis on the social significance of behavior and the magnitude of its change (Baer et al., 1968; Lattal & Laipple, 2003). In the prior definition of the science, both the focus on machine behavior and the relevance of such behavior underlie this pragmatic perspective. A science of machine behavior analysis is relevant if it promotes effective action on the behavior of the user for which the machine was designed. By sharing philosophical assumptions with the other domains of behavior analysis, machine behavior analysis may produce results that are coherent and consistent with the sciences that focuses on living organisms.

This reliance on the philosophical underpinnings of behavior analysis should not restrict, or limit, interdisciplinary collaborations. On the contrary, a science of machine behavior analysis will most likely owe its success to fruitful collaborations with other natural scientists and engineers. For example, natural sciences and engineering share many of our epistemological positions regarding determinism, empiricism, parsimony, and pragmatism. Moreover, several behavioral terms have already made their way into the engineering of biological and nonbiological machines (e.g., shaping, schedules of reinforcement, classical conditioning, operant conditioning; Abramson & Levin, 2021; Kaelbling et al., 1995; Konidaris & Barto, 2006; Zhang et al., 2020). Working in interdisciplinary teams may not only improve the scope and depth of research in machine behavior analysis, but it may also support the survival of the verbal community in the long term. The interdisciplinary approach has already supported our field in the past: interdisciplinarity collaborations in the treatment of autism and in the study of delay discounting have both contributed to the development and promotion of applied behavior analysis and the experimental analysis of behavior, respectively (Raches et al., 2019; Reynolds, 2006; Roane et al., 2016).

## Some Arguments in Favor

To summarize, machine behavior analysis is a science that examines how machines interact with and produce relevant changes in their external environment by relying on replicability, behavioral terminology, and the philosophical assumptions of behavior analysis (e.g., selectionism, determinism, parsimony) to study artificial behavior. Although this definition provides some boundaries for machine behavior analysis, a logical follow-up question is: Why should behavior analysts care about machine behavior? The next sections present arguments in support of encouraging more research on machines from a behavior analytic perspective and provide some examples of relevant areas of research.

### Machines Are Here to Stay and Just Keep Getting Better

Machines are an increasing part of every domain of our daily lives. In 2015, there was approximately two connected devices per human on earth (Safaei et al., 2017). Safaei et al. estimated that the number of connected devices would increase to nine per human on earth by 2030. Likewise, researchers expect that the amount of electronic waste (e.g., broken smartphones, obsolete computers) generated by humans will more than double between 2011 and 2030 (Shittu et al., 2020). Both prior estimates were produced prior to the COVID-19 pandemic, which has only accelerated the adoption of electronic machines in multiple domains of our lives (Vargo et al., 2021). That said, an increase in the number of machines alone may not justify the study of their behavior. As discussed previously, studying predictable machine behavior has limited relevance. The issue is that the number of machines has not only increased, but these machines are also getting much “smarter” and should keep doing so in the foreseeable future (Arif Wani et al., 2020; Hayhurst, 2019; Mammela & Anttonen, 2017). Said differently, both the amount of data taken as input and the algorithmic complexity of machines are increasing over time, which makes it more and more difficult to explain why they exhibit one specific response over another.

Some propositions for the future may push the limits of what machines may do and how they differ from humans. For example, researchers have recently developed a culturally competent robot that improves emotional well-being in older adults when compared to treatment as usual (Papadopoulos et al., 2021). In time, such robots may take care of both the physical and psychological needs of older adults (e.g., Bardaro et al., 2021; Costa et al., 2018; Niemelä & Melkas, 2019; Papadopoulos et al., 2021). In a more ambitious vein, the Alan Turing Institute (2020) proposed the AI Scientist Grand Challenge, which aims to develop a machine that could win a Nobel prize in science by 2050. These more complex machines mean that engineers are not necessarily able to predict whether a machine will exhibit one behavior rather than another (or not at all) when provided with a specific input (von Eschenbach, 2021; Wadden, 2021). Given recent developments in artificial intelligence, the question is not if, but when machines will be able to have interactions that are indistinguishable from humans. Thus, behavior analysts should begin studying the behavior of machines now and develop tools to do so as they will become more complex in the future.

### Machines Are Already Changing Human Behavior

A basic assumption of behavior analysis is that the environment evokes, elicits, or selects human behavior, and machines are already changing behavior. Amongst the most popular machines with which humans interact daily, Amazon recommends products based on prior purchases, Google autocompletes search queries and provides results based on history, Facebook relies on what people like to suggest news that they should watch, and Alexa adapts its responses to human interactions. Therefore, machines can have a socially significant impact on behavior for better (e.g., recommending a mental health provider to someone who displays behaviors associated with depression) or worse (e.g., recommending a news outlet that promotes bigotry or unvalidated treatments).

The applied and experimental sciences already study the interaction between humans and machines by focusing mainly on the behavior of the former (e.g., Critchfield & Perone, 1990; Dallery et al., 2021; Higbee et al., 2016). This reality begs the question, why should the field have a science of machine behavior analysis if we already study interactions between humans and machines? The response is that there are many situations in which behavioral researchers may want to isolate machine, rather than human, behavior. In these situations, behavioral researchers reverse the role of each variable in their inquiry. The machine behavior becomes the dependent variable whereas the human behavior is the independent variable. This approach contrasts with traditional studies with machines in behavior analysis wherein the dependent variable is the human behavior and the machine functions as the independent variable. For the same reasons that experimental analysts study behavior in the laboratory to better control for the effects of confounding variables, studying machine behavior on its own appears essential to contribute to our understanding of its interaction with its external environment.

### Engineers Alone Do Not Have all the Answers

In general, behavioral researchers perceive machine behavior as being the concern of engineers. Engineers have a clear and central role in machine behavior: they are the ones who develop the hardware and code the software that the machines use to behave. However, technology is reaching a point where engineers are unable to predict what behavior a machine will engage in following specific inputs, which is even leading to a crisis as to whether machines should be trusted with important decisions (von Eschenbach, 2021; Wadden, 2021; Wiens et al., 2019). Moreover, engineers are trained to change machine behavior through coding, but are not specialized in modifying the physical and social environment to alter behavior. Because machine behavior may also be modified by altering the environment (other than coding), society needs a science beyond computer engineering to investigate machine behavior.

Behavior analysts are uniquely trained and positioned to address both the previous issues. First, the field has developed expertise and methodology in studying a subject that has a similar input (environment) and output (behavior). Likewise, behavioral researchers may look beyond the algorithms and coding (as with the brain and nervous system) and examine how the environment affects machine behavior. Second, behavior analysts are experts in modifying behavior using the social and physical environment. As machines are being increasingly designed to interact with humans in a manner similar to other humans, behavioral researchers may apply their knowledge and experience to modify machine behavior without the use of coding. This involvement may eventually lead to machines responding in a manner that is more beneficial to its users.

This argument does not aim to exclude engineers from studying and contributing to a science of machine behavior. When studying human behavior, many covert events (e.g., verbal behavior, imagery) remain inaccessible and unmanipulable to the experimenter. This inaccessibility issue does not apply to machine hardware and software, which is a major difference between machines and living organisms. Even though engineers may be unable to predict how specific environmental changes may affect the responding of their machine, they can still modify the machine rather than the environment to change behavior. This reality supports the relevance of studying machine behavior using an interdisciplinary approach. Under ideal conditions, behavioral researchers and engineers should work together to address important questions involving machine behavior. Engineers can manipulate the machine itself (i.e., hardware and software) whereas behavioral researchers may support them in studying how changes to the environment affects behavior. This synergy should accelerate and contribute to the knowledge base in both fields while improving machines for the benefit of humankind.

### A Science Requires an Organized Community

One alternative to formalizing a science of machine behavior analysis is to incorporate it within an existing domain of behavior analysis, such as the experimental analysis of behavior or applied behavior analysis. One problem with this integration is that the focus of these domains is the behavior of living organisms. At this point, researchers have no reason to believe that all machines will behave in the same way as humans, or other living organisms, in the presence of specific environmental stimuli. Although the philosophy and methodologies of behavior analysis appear relevant to a science of machine behavior analysis, the processes and patterns uncovered for machines may differ considerably from cell-based organisms. As such, having a separate domain would allow for the eventual development of a knowledge base specific to machines.

The 20^th^ century has seen the development of many theories of knowledge to explain what constitutes a science such as Popper’s falsification (1934/2002), Kuhn’s paradigm shift (1962/2012), and Rorty’s perspective on pragmatism (1979/2017). One commonality between these epistemologies is that a science develops within an organized community. To develop and to grow, sciences must be organized around a community who intersubjectively debate, discuss and agree on facts and ideas. In more behavior analytic terms, a science constitutes a verbal community that shares rules and contingencies acquired through contact with the environment (i.e., experiences and training). When the words, rules, and contingencies among domains differ sufficiently, a new science typically emerges. The position of this article is that machine behavior differs enough from the behavior of living organisms to at least discuss the relevance of having its own domain.

To be clear, researchers have already conducted studies that meet most, if not all, the characteristics described previously. More than 20 years ago, Saksida et al. (1997) proposed using the principles of reinforcement to condition and shape robot behavior, albeit from an engineering standpoint. In the same year, Burgos (1997) discussed the training of artificial networks involving Pavlovian conditioning processes. Put differently, some researchers are already contributing to machine behavior analysis, even though they may not refer to it as such. From philosophical and theoretical standpoints, behavioral researchers have speculated on important questions regarding machines such as whether a machine can be made “human” (Hutchinson, 2012; Rachlin, 2012) and whether having a nervous system is a necessary precondition for learning (Burgos, 2018). From an empirical standpoint, behavior analytic journals have published several studies simulating the behavior of machines and how they compared to the behavior of living organisms (e.g., Burgos, 2007; Lyddy et al., 2002; McDowell, 2004, 2019; Ninness & Ninness, 2020; Vernucio & Debert, 2016). These examples are not exhaustive as the purpose of this article was not to provide a systematic review of prior research in machine behavior analysis. The proposal here is to organize and formalize the science within a community to accelerate and promote the development of this research domain.

### To Simulate Organisms

As the behavioral literature contains numerous examples of simulations, I will take a concrete example to illustrate the characteristics and importance of having a science of machine behavior. To this end, the current section will focus on a study published by McDowell (2004) entitled, “A Computational Model of Selection by Consequence.” In his study, McDowell created a digital organism programmed using the evolutionary algorithm, which was then subjected to a random-interval schedule of reinforcement. Two of the main findings were that (1) the digital organism’s responding closely followed the hyperbolic form of the quantitative law of effect (i.e., single-alternative matching equation) and (2) responding under certain conditions remained consistent with patterns observed in rats from prior research (Dallery et al., 2000).

Before discussing the relevance of such research, let’s examine whether the study meets the five characteristics of a science of machine behavior analysis. First, the study involves a machine behavior: printing numbers on a screen. Second, this machine behavior is relevant to a behavioral scientist who aims to examine its patterns of responding. That is, examining the machine behavior directly alters the behavior of the scientist when developing and testing hypotheses and theories. Third, McDowell (2004) provides sufficient details for replication while using methods that can produce reproducible results. The methods of analyses used by McDowell are also common in the experimental analysis of behavior. The fourth characteristic involves remaining consistent with behavior analytic terminology. The author uses common terminology and concepts from behavior analysis such as fixed ratio 1 schedule, random-interval schedule, reinforcement rate, and responses rate. Finally, the article addresses and remains grounded in many philosophical underpinnings of behavior analysis. The title underlines the focus of the article on selectionism, the simulation and discussion rely on determinism to explain machine behavior, the study involves empirical methods, and explanations of potential mechanisms for change remain parsimonious. In sum, the study would meet all the defining characteristics of a science of machine behavior analysis as proposed in the current article.

This analysis leads to an important question: Why should behavioral researchers simulate organisms in the first place? The first answer to this question is discussed by McDowell (2004). Simulating organisms may assist researchers in testing models or patterns of behavior that have been observed in living organisms. In general, because machine responding is more stable than that of living organisms, it may be possible to remove some of the noise to validate one model, or hypothesis, over another. By simulating an organism’s behavior and observing patterns in responding, McDowell provided further support for the hyperbolic matching equation, which had been developed with living organisms. The second answer to the question involves experiments that would be difficult or impossible to conduct with living organisms. For example, a researcher may aim to examine how behavioral contingencies in one generation of organisms influence responding in future generations while interlocking contingencies (metacontingencies; Glenn, 1988) are operating. Studying more than a few generations of an organism may be a challenge in the laboratory and the same can be said of investigating many organisms simultaneously interacting within the same environment. Simulating organisms addresses these limitations as computer simulations allow for the study of hundreds of digital organisms interacting together, which can be extended over numerous generations. Such an endeavor could inform researchers on how interlocking contingencies and complex systems influence behavior within a behavior analytic framework. Simulating organisms thus opens innovative avenues for research in the field of behavior analysis.

### To Study Machine Behavior

Another area of research to which behavioral scientists could contribute is the study of machine behavior. To describe what this approach may entail, I will discuss the development of socially assistive robots for people with autism as an example. Dickstein-Fischer et al. (2018) proposed developing robots that could improve accessibility to behavioral interventions in this population. For example, a simple robot could reinforce appropriate play behavior of a child with autism using a shaping procedure. If the robot is prepared using machine learning, engineers would initially train it by providing video exemplars of children playing and not playing. Once the engineers have developed and trained the robot, behavioral researchers could test the robot in generalized settings to examine how it responds and adapts to novel situations. The dependent variable could involve the number of steps implemented correctly in an integrity treatment checklist for shaping. In baseline, the experimenter could ask a confederate to wait quietly in a room while ignoring the robot. In this case, we would not expect the robot to display behavior from the treatment integrity checklist. In the intervention, the confederate could follow a script by playing a game in an increasingly appropriate manner (with some occasional relapses) to see how the robot reacts. This process could be repeated across different confederates or different play behaviors within a multiple baseline design. It is important to stress that the dependent variable in this study is the percentage of treatment integrity steps completed correctly by the machine and *not* the play behavior of the confederate.

This experiment would show whether the robot implements shaping correctly in a real-world environment. This step is essential to validate that the machine is safe and produces the expected response patterns given real-world inputs prior to conducting applied studies with children with autism. If the robot is not performing shaping correctly, engineers and behavioral researchers should go back to improve the machine and test it again within a contrived environment. Once the machine displays behavior consistent with its intended functioning, studies can be conducted with children with autism. The locus of analysis would now change from machine behavior to child behavior, which falls within applied behavior analysis. This sequence moving from machine behavior analysis to applied behavior analysis would reduce potential harm by making sure that the machine performs as intended before conducting applied studies. Hence, behavioral researchers have an important role to play in studying the behavior of machines.

### To Modify Machine Behavior

Some machines learn and adapt their responding to their environment. Tay the chatbot is good example of such a machine, which adapted its responding to what others were saying on Twitter. Engineers may struggle in dealing with the unpredictability of machine learning using programming alone without compromising other functions or characteristics of Tay. One solution is for behavioral researchers, rather than engineers, to modify the behavior of machines that “misbehave.” In the coming decades, machines will only get more complex and may begin learning more from their environment than from the data that was provided initially by the engineer. In other words, the environment may eventually have a stronger effect on machine behavior than the initial coding. Behavior analysts will be well-positioned to change the behavior of these machines by manipulating their external environment.

Imagine that a long-term care facility purchases a robot designed to socialize with older adults (e.g., Bardaro et al., 2021). After a few weeks in the long-term care facility, the staff report that they find it disconcerting that the robot only interacts with the older adults, but never responds to the staff. The staff would like the robot to at least acknowledge their presence when they enter a room. If the behavior of the robot can be modified through reinforcement and shaping, a behavior analyst could manipulate the environment so that the robot begins also interacting with care staff. In this case, the machine does not need to be recoded by an engineer; instead, a behavior analyst sets up novel environmental contingencies that teaches the robot to generalize responding to younger individuals. In more general terms, a behavioral researcher could manipulate the environment to evoke the target behavior under the expected stimulus conditions when a “learning” machine behaves in an unexpected or undesirable manner. This training could involve single-case experimental designs to identify the variables that control the target behavior akin to many studies in the experimental and applied domains. Once the behavior analyst has trained the machine to behave in the desired manner, the engineer can clone the state of the machine to replicate it. In this example, behavior analysts do not limit themselves to studying machine behavior: they actively contribute to modifying it through the manipulation of the environment for which they are experts.

## Some Future Directions

Most exemplars of machine behavior analysis from the behavior analytic literature involve experimental work to simulate models of behavior. Behavioral researchers may also study machines that already exist or participate in the development of novel machines. One potentially fertile area for future research is the study of machines designed to shape consumer behavior. Every day, hundreds of millions of consumers use server-based applications such as Facebook, Netflix, Spotify, Amazon, TikTok, and Twitter. Because the code and algorithms of these apps are proprietary, researchers have limited knowledge on how these machines respond to human input. Even if researchers had access to the source code, predicting specific responses would probably be unrealistic, or even impossible, without conducting an empirical study to examine the effects of specific input on machine behavior. In the prior example, interdisciplinary teams including behavioral researchers may work together and conduct empirical studies to uncover how the environment shapes the machine’s behavior. Such an approach could involve a combination of research methods from engineering as well as behavior analysis. As behavioral shaping between machine and human can be a two-way interaction, studying how these machines respond to human behavior appears essential to improve their potential effects (Bucher, 2017).

Another area of research is health recommendation systems. Researchers are increasingly developing machines to support clinical decision making (Wiens et al., 2019). One behavior analytic exemplar is a tool designed by Lanovaz et al. (2020) to determine whether an AB graph shows a clear change. This web app may be used by mental health professionals to decide whether to continue or interrupt a behavioral intervention. The machine may take better decisions than humans, but a problem that remains is that researchers and practitioners have no idea what type of decision errors this machine makes. To examine this question, researchers need to study the machine’s behavior in isolation when given different inputs. Recommendation systems may even adapt their responding over time based on human responding (Derakhshan et al., 2019), underlining the relevance of the topic for behavior analytic research.

Machines may also be used to personalize teaching strategies to a learner’s characteristics and responding (Luan & Tsai, 2021). The issue is that some of the decision-making algorithms are proprietary and other algorithms function as black boxes, making it nearly impossible for a human observer to predict its behavior based on code alone. Before using these types of applications in practice or research, behavioral researchers may conduct studies to examine how the machine responds to different patterns of input. Systematically studying machine behavior is one way to identify how these machines will interact with us and clients, which may ultimately lead to better decisions when selecting one technological alternative over another. In terms of ethics, behavior analysts may also need to understand how a learning application operates prior to the implementing it with their students.

Engineers are developing machines that are increasingly designed to behave and interact like humans. As noted earlier, some behavioral researchers have even developed models that exhibit patterns similar to those observed in humans (e.g., Burgos, 2007; Lyddy et al., 2002; Ninness & Ninness, 2020; Vernucio & Debert, 2016). Two categories of machines that are often designed to behave like humans are chatbots and social robots. Thus, one fruitful area of research could be to study the verbal behavior of chatbots. How do chatbots respond to a human given different antecedents and consequences? Likewise, behavioral researchers may study the behavior of social robots who are designed to interact with, or even replace, humans in complex tasks. Manipulating the environment to examine how they react to changes is essential to ensure that these robots actually engage in behavior beneficial to humans. It should be noted here that the possibilities to study machine behavior are endless. The previous directions for future research are provided as examples as to how a science of machine behavior analysis could contribute to the advancement of behavior analysis.

The use of machines to deliver educational and health services raises important issues related to the ethical, legal, and professional oversight of machine behavior. If a family chooses a teaching app that relies on machine learning for their child and this app proves ineffective or produces an undesirable side-effect, who is ethically and professionally liable for the machine’s behavior? What happens if the machine’s behavior was partly shaped by a behavior analyst? Does the behavior analyst have ethical and professional obligations towards every user of the app? Can a user file a complaint to their certification or licensing board? I do not have an answer to these questions, but these issues will have to be addressed in the years to come. The responsibility for machine behavior is an urgent issue that researchers need to consider promptly. This issue is not limited to machine behavior analysis: Both the applied and practice domains are at the frontlines of using machines in health care and education. A future direction should involve a collaboration between different domains of behavior analysis as well as engineers to tackle these important questions before the science moves forward.

## Conclusion

Skinner (1969) argued that “man is a machine, but he is a very complex one” (p. 294). The proposal for a science of machine behavior analysis remains consistent with this original conceptualization of humans. At the time, machine behaviors were probably not complex enough to warrant their own domain. With the evolution of computing power and algorithms, researchers have already reached a point where an understanding of engineering is insufficient to explain and predict all machine behavior. More important, the field needs a debate on the topic, regardless of whether behavior analysts agree or disagree with this proposal. Does behavior analysis need a fifth domain? Do the proposed characteristics bound the science adequately? There is no doubt that behavioral researchers are already conducting studies and having academic discussions on the topic. The current article aims to encourage and contribute to this debate. Ultimately, a science of machine behavior analysis may help shape the behavior of machines to better meet the needs of humanity.

## Footnotes

1 To improve the flow of the article, I use the generic term “engineer” to refer to any professional who develops hardware, software, and algorithms for machines (including computer scientists).
